# Molecular and Functional Characterization of Three Different Postzygotic Mutations in *PIK3CA*-Related Overgrowth Spectrum (PROS) Patients: Effects on PI3K/AKT/mTOR Signaling and Sensitivity to PIK3 Inhibitors

**DOI:** 10.1371/journal.pone.0123092

**Published:** 2015-04-27

**Authors:** Daria C. Loconte, Valentina Grossi, Cristina Bozzao, Giovanna Forte, Rosanna Bagnulo, Alessandro Stella, Patrizia Lastella, Mario Cutrone, Francesco Benedicenti, Francesco C. Susca, Margherita Patruno, Dora Varvara, Aldo Germani, Luciana Chessa, Nicola Laforgia, Romano Tenconi, Cristiano Simone, Nicoletta Resta

**Affiliations:** 1 Division of Medical Genetics, Department of Biomedical Sciences and Human Oncology (DIMO), University of Bari ‘Aldo Moro’, Bari, Italy; 2 National Cancer Institute, IRCCS Oncologico Giovanni Paolo II, Bari, Italy; 3 Department of Clinical and Molecular Medicine, "Sapienza" University of Rome, Rome, Italy; 4 Cancer Genetics Laboratory, IRCCS “S. de Bellis”, Castellana Grotte, Italy; 5 Center for Rare Diseases-Internal Medicine "C. Frugoni", University Hospital of Bari, Bari, Italy; 6 US Dermatologia Pediatrica, Ospedale dell'Angelo Ulss 12 Mestre, Venezia, Italy; 7 Genetic Counseling Service, Department of Pediatrics, Regional Hospital of Bolzano, Bolzano, Italy; 8 Neonatology and NICU Section, Department of Biomedical Sciences and Human Oncology (DIMO), University of Bari ‘Aldo Moro’, Bari, Italy; 9 University of Padova, Padova, Italy; Peter MacCallum Cancer Centre, AUSTRALIA

## Abstract

**Background:**

*PIK3CA*-related overgrowth spectrum (PROS) include a group of disorders that affect only the terminal portion of a limb, such as type I macrodactyly, and conditions like fibroadipose overgrowth (FAO), megalencephaly-capillary malformation (MCAP) syndrome, congenital lipomatous asymmetric overgrowth of the trunk, lymphatic, capillary, venous, and combined-type vascular malformations, epidermal nevi, skeletal and spinal anomalies (CLOVES) syndrome and Hemihyperplasia Multiple Lipomatosis (HHML). Heterozygous postzygotic *PIK3CA* mutations are frequently identified in these syndromes, while timing and tissue specificity of the mutational event are likely responsible for the extreme phenotypic variability observed.

**Methods:**

We carried out a combination of Sanger sequencing and targeted deep sequencing of genes involved in the PI3K/AKT/mTOR pathway in three patients (1 MCAP and 2 FAO) to identify causative mutations, and performed immunoblot analyses to assay the phosphorylation status of AKT and P70S6K in affected dermal fibroblasts. In addition, we evaluated their ability to grow in the absence of serum and their response to the PI3K inhibitors wortmannin and LY294002 *in vitro*.

**Results and Conclusion:**

Our data indicate that patients’ cells showed constitutive activation of the PI3K/Akt pathway. Of note, PI3K pharmacological blockade resulted in a significant reduction of the proliferation rate in culture, suggesting that inhibition of PI3K might prove beneficial in future therapies for PROS patients.

## Introduction

In 1997 Cynthia A. Moore *et al*. [1] described a new sporadic overgrowth disorder with a combination of anomalies including macrocephaly, megalencephaly, cutis marmorata telangiectatica congenita and foot abnormalities different from the condition known as cutis marmorata telangiectatica congenita.

In the following years, two megalencephaly (MEG) syndromes were recognized: the megalencephaly-capillary malformation (MCAP) syndrome, formerly called macrocephaly-capillary malformation (MCM) syndrome, characterized by involvement of the CNS, growth dysregulation with body asymmetry (hemihyperplasia), vascular anomalies and distal limb malformations (polydactyly and syndactyly), and the closely related megalencephaly-polymicrogyria-polydactyly-hydrocephalus (MPPH) syndrome, which lacks the vascular malformations and syndactyly of MCAP. These two easily recognizable and sporadic conditions show similar brain involvement with (hemi)megalencephaly, ventriculomegaly, polymicrogyria, and cerebellar tonsillar ectopia progressing to Chiari anomaly; this suggests that they are due to *de novo* mutations of genes of the same pathway [2]. So far, *PIK3CA* postzygotic mutations have been described in almost all cases of MCAP/MPPH, while mutations in the *AKT3* and *PIK3R2* genes have been detected in a few cases [2]. Very recently, *de novo* heterozygous activating mutations in the *CCND2* gene (encoding cyclin D2) were identified in MPPH patients lacking upstream PI3K/AKT pathway mutations [3].

Postzygotic mutations in the *PIK3CA* gene have also been identified in distinct overgrowth syndromes such as CLOVES (congenital lipomatous asymmetric overgrowth of the trunk, lymphatic, capillary, venous, and combined-type vascular malformations, epidermal nevi, skeletal and spinal anomalies), HHML (Hemihyperplasia Multiple Lipomatosis) and fibroadipose overgrowth (FAO) [4–6]. CLOVES syndrome differs from MCAP syndrome for a more marked growth dysregulation, with lipomatous tissues showing complex congenital overgrowth (typically appearing as a truncal lipomatous mass) and a combination of vascular and lymphatic malformations. FAO shares clinical and molecular features with CLOVES syndrome and may involve the trunk or extremities. It is characterized by progressive segmental overgrowth in various regions of the body including visceral, subcutaneous, muscular, fibroadipose, and skeletal tissues.

Recently, for all these clinical entities characterized by the presence of activating somatic mutations in the PIK3CA gene, the new term of PIK3CA-Related Overgrowth Spectrum (PROS) has been proposed so to comprehend the broad range of clinical manifestations in these patients [7].

To identify causative mutations in three patients with clinical symptoms consistent with PROS, we performed Sanger sequencing and targeted deep sequencing of 21 selected genes involved in the PI3K/AKT/mTOR pathway in three patients, one affected by MCAP, and two by FAO. In three of them we identified a causative mutation in the PIK3CA gene, which encodes the p110α catalytic subunit of the phosphoinositide-3-kinase heterodimer. Moreover, we evaluated the phosphorylation status of AKT and P70S6K in primary affected dermal fibroblasts and assessed cell growth upon treatment with PI3K inhibitors.

## Materials and Methods

### Patient recruitment

All patients signed an informed consent approved by the local ethics committee to participate in this study and to authorize the publication of clinical images. Blood and tissue samples were collected during surgical debulking procedures performed for the treatment of FAO.

### DNA Extraction and Sanger Sequencing

Genomic DNA was extracted from peripheral blood cells (PBCs) and tissue samples using the QIAamp Mini Kit (Qiagen, Hilden, Germany), according to the manufacturer’s instructions, and quantified on a BioSpectrometer Plus (Eppendorf, Hamburg, Germany). The entire coding regions of *PIK3CA* (RefSeq NM_006218.2), including all splice junctions and adjacent intronic sequences were amplified by standard PCR protocols using the AmpliTaq Gold DNA Polymerase (Applied Biosystems, London, UK) and the primer pairs listed in Table A in S1 File. Direct sequencing was performed using the BigDye Terminator v1.1 Cycle Sequencing Kit (Applied Biosystems) according to the manufacturer’s instructions on an ABI 310 Genetic Analyzer (Applied Biosystems).

### Targeted Deep Sequencing

21 genes involved in the PI3K/AKT/mTOR pathway (*PIK3R1*, *PIK3R2*, *PIK3CA*, *PTEN*, *PDK1*, *PDK2*, *KRAS*, *AKT1*, *AKT2*, *AKT3*, *RICTOR*, *MAPKAP1*, *MLST8*, *MTOR*, *IRS1*, *GAB1*, *GAB2*, *THEM4*, *MAPK8I1*, *PTPN11*, *RAPTOR*) were selected for targeted sequencing, see **Table B in** S1 File. An Ion AmpliSeq Custom Panel was designed online using Ion AmpliSeq Designer 2.2 (http://www.ampliseq.com/) to analyze the CDSs (+/-25 bp of intronic flanking regions) of these genes. The final custom panel was composed of 554 amplicons divided into 2 primer pools for a total of 66.58 kb of DNA. DNA was quantified using the Qubit dsDNA HS Assay Kit (Life Technologies) on a Qubit2.0 Fluorometer (Life Technologies).

The panel covered 97.45% of the regions of interest (ROI). Libraries were prepared using the Ion AmpliSeq Library Kit v2.0 (Life Technologies), according to the manufacturer's instructions. One of 16 barcodes of the Ion Xpress Barcode Adapters 1–16 Kit (Life Technologies) was added to each sample. Libraries were quantified with the Qubit dsDNA HS Assay Kit (Life Technologies) on a Qubit2.0 Fluorometer (Life Technologies) and equimolar amounts of each library were used to prepare templates for clonal amplification. Emulsion PCR was performed on a OneTouch2 system (Life Technologies) using the Ion PGM Template OT2 200 Kit (Life Technologies). Templates were enriched using Ion OneTouch ES (Life Technologies) and prepared for loading on a 316v2/318v2 chip. Groups of 4 sample libraries were sequenced on each chip. Sequencing runs were performed on an Ion Torrent Personal Genome Machine (Life Technologies) using the Ion PGM Sequencing 200 Kit v2 (Life Technologies), according to the manufacturer’s instructions.

### Alignment

Data analysis was performed using Torrent Suite Software v.4.0.2 (Life Technologies). Reads were aligned to the hg19 human reference genome from the UCSC Genome Browser (http://genome.ucsc.edu/) and to the BED file designed using Ion AmpliSeq Designer. Alignments were visually verified with the Integrative Genomics Viewer (IGV) v.2.3 (www.broadinstitute.org/igv/home).

### Coverage Analysis

The mean average read depth and the percentage of reads that mapped on the ROI out of the total number of reads (reads on target) were calculated using the Coverage Analysis plugin (Torrent Suite 4.0 software, Life Technologies). For each sample, the percentage of ROI with a minimum coverage of 200X was calculated using the amplicon coverage matrix file.

### Variant Analysis

Variant calling was performed with the Variant Caller plugin configured with somatic high stringency parameters. Variants were annotated using the Ion Reporter 4.0 software (https://ionreporter.lifetechnologies.com/ir/). Common single nucleotide variants (minor allele frequency [MAF] >5%), exonic synonymous variants and intronic variants were removed from the analysis, while exonic non-synonymous, splice site and loss-of-function variants were analyzed. The pathogenicity prediction programs PolyPhen2 and SIFT and splice prediction programs were used to evaluate variants not previously described.

### Cell Culture and Reagents

IMR90 human primary fibroblasts (from ATCC) were grown in DMEM supplemented with 10% FBS, 100 IU/ml penicillin and 100 μg/ml streptomycin; patients-derived primary fibroblasts were grown in RPMI supplemented with 10% FBS, 100 IU/ml penicillin, 100 μg/ml streptomycin and 1% L-glutamine in a humidified incubator at 37°C and 5% CO_2_ avoiding confluence at any time. Wortmannin (10 μM) and LY294002 (25μM) were purchased from Sigma-Aldrich (Poole, UK) and Selleckchem (Houston, TX), respectively.

Skin biopsies were washed in PBS, then transferred in a 35-mm petri plate containing 2 ml of serum free D-MEM. The tissue was minced into fine fragments using a scissor, and subsequently transferred in a 15-ml centrifuge tube containing 2 ml of 1 mg/ml Collagenase II dissolved in serum free D-MEM.

After 2–3 hours at 37°C in a CO_2_ incubator, dissociated clumps of cells and loose cells were washed in cold serum free D-MEM to remove Collagenase and finally transferred in a 25-cm^2^ culture flask with 5 ml of complete D-MEM medium (15% Fetal Calf Serum, 1x L-Glutamine, 1x Penicillin/streptomycin) and incubated in CO^2^-incubator.

After 3–4 days of culture, when cellular growth became evident, medium was replaced twice per week. When several colonies were well expanded with rounded up mitotic cells and 60–70% confluence was reached, subcultures were set up splitting primary culture by Trypsin-EDTA solution cell detachment.

Molecular studies were performed on cell cultures obtained from the split of primary cultures (first subculture, 1^st^ passage) or on cell cultures obtained from the splitting of confluent first subcultures (2^nd^ passage).

### Quantification of Cell Number

The reported number of primary cells was determined by counting. Supernatants (containing dead/floating cells) were collected, and the remaining adherent cells were detached by Trypsin/EDTA (Sigma-Aldrich). Cell pellets were resuspended in 1X PBS and 10 μl were mixed with an equal volume of 0.01% trypan blue solution. Viable cells (unstained, trypan blue negative cells) and dead cells (stained, trypan blue positive cells) were counted with a phase contrast microscope.

### Cell Proliferation Assay (WST-1)

Cell proliferation was determined using the Cell Proliferation Reagent WST-1 (Roche, Mannheim, Germany) as per manufacturer’s instructions. Briefly, cells were seeded into 96-well plates one day before treatment. After 12h, 24h, 36h or 48h of drugs (or DMSO) exposure, 10 μl of the Cell Proliferation Reagent WST-1 were added to each well and incubated at 37°C in a humidified incubator for 1h. The absorbance was measured on a microplate reader (BioTek, Seattle, USA) at 450/655 nm. Each assay was performed in 6 replicates and the experiment was repeated six times. The proliferation index was calculated as the ratio of WST-1 absorbance of treated cells at the indicated time point (12h, 24h, 36h or 48h) to WST-1 absorbance of the same experimental group at 0h.

### Immunoblot Analysis

Immunoblotting analyses were performed according to Cell Signaling Technology instructions (Beverly, USA). Briefly, cells were homogenized in 1X lysis buffer (50 mM Tris-HCl pH 7.4; 5 mM EDTA; 250 mM NaCl; 0.1% Triton X-100) supplemented with protease and phosphatase inhibitors (1 mM PMSF; 1.5 μM pepstatin A; 2 μM leupeptin; 10 μg/ml aprotinin, 5 mMNaF; 1 mM Na_3_VO_4_). 15 to 20 μg of protein extracts from each sample were denatured in 5x Laemmli sample buffer and loaded into an SDS-polyacrylamide gel for western blot analysis. Western blots were performed using polyclonal anti-β-Actin (Sigma-Aldrich; Cat N° A 2066; dil: 1:10000; rabbit; antigen: C11 [11 aa in C- terminal]), monoclonal anti-phospho-Akt (Thr308) (Cell Signaling Technology; Cat N° #2965, dil: 1:250; rabbit; antigen: synthetic phospho-peptide [KLH-coupled] corresponding to residues around Thr308 of a mouse Akt), polyclonal anti-phospho-Akt (Ser473) (Cell Signaling Technology; Cat N° 9271; dil: 1:250; rabbit; antigen: synthetic phospho-peptide [KLH-coupled] corresponding to residues around Ser473 of a mouse Akt), polyclonal anti-Akt (Cell Signaling Technology; Cat N° #9272; dil: 1:500; rabbit; antigen: synthetic peptide [KLH-coupled] derived from carboxy-terminal sequence of a mouse Akt), polyclonal anti-phospho-p70S6K (Ser371) (Cell Signaling Technology; Cat N° #9208; dil: 1:250; rabbit; antigen: synthetic phospho-peptide [KLH-coupled] corresponding to residues around Ser371 of humane p70S6 kinase). Western blots were developed with the ECL-plus chemiluminescence reagent (GE Healthcare, Uppsala, Sweden) as per manufacturer's instructions.

### Statistical Analysis

Statistical significance of the results was analyzed using Student’s t-test. *P*<0.05 was considered statistically significant.

## Results

### Patients and clinical findings

#### Patient 1

**Fig 1:**
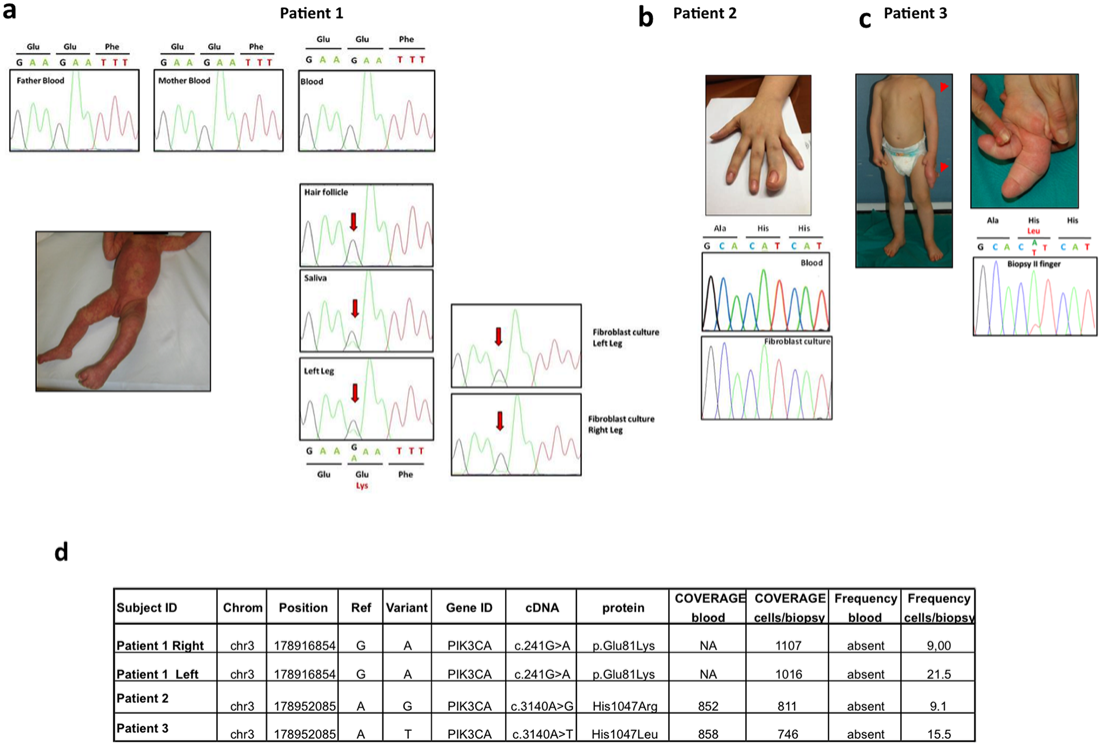
Clinical and mutational spectrum of the three index cases. **a** Patient 1, clinically diagnosed with MCAP, showing diffuse capillary malformation at the age of 2 months and cutaneous syndactyly between the 2^nd^ and 3^rd^ toes. The *PIK3CA* c.241 G>A [p.E81K] mutation detected by Sanger sequencing in affected cells and tissues of patient 1 showed varying levels of the mutant allele depending on the tissue tested. The mutation was absent in the patient's blood and in her parents. **b** Macrodactyly of the right 4^th^ finger in patient 2, diagnosed with FAO, at the age of 17 years. Sequence of *PIK3CA* exon 20 in blood and cultured fibroblasts obtained from patient 2 showing that the mutation is undetectable in these samples. **c** Patient 3, at the age of 15 months before surgical intervention; note the disproportion of the left 2^nd^ and 3^rd^ fingers and the subcutaneous mass at the left deltoid region. Sanger sequencing validation of the c.3140 A>T [p.H1047L] mutation detected with targeted deep sequencing in the biopsy from the 2^nd^ finger of patient 3. **d** List of samples and mutations detected with targeted deep sequencing. Coverage indicates the mean average of reads on target in the regions of interest (ROI) while frequency denotes the percentage of reads with the mutation.

Patient 1, a female aged 10 years and 4 months, is the first of two children of a healthy 38-year-old woman and a non-consanguineous 48-year-old man, whose family history was unremarkable. She was conceived naturally. The fetal ultrasound scan at gestational week (gw) 12 was normal, while at gw 21 a choroid plexus cyst and echogenic intracardiac focus were detected, the biparietal diameter was at the 95^th^ centile at gw 21 and >95^th^ centile at gw 33 with normal cerebral morphology. She was born at the 34^th^ gw with a weight of 3080 g (90-97^th^ centile), length of 49 cm (90^th^ centile) and head circumference of 35.6 cm (97^th^ centile). At birth, a diffuse capillary malformation involving the trunk and limbs was observed, associated with cutaneous syndactyly of the 2^nd^ and 3^rd^ toes (Fig 1a). During the first year of life, a plantar epidermal nevus and a small hemangioma, which subsequently disappeared, were observed, together with left hemihyperplasia (detected at the age of 3 months).

Cardiac and abdominal ultrasound scans were repeatedly normal. Brain MRI scans detected increased peritrigonal signal of white matter at the age of 10 months and focal hemimegalencephaly with perisylvian polymicrogyria at the age of 7 years. From the age of 7 years, the girl had episodes of generalized tonic-clonic seizures refractory to antiepileptic therapy. She suffers from mild cognitive impairment and attention deficit disorder, and in the last years had temper tantrums and angry outbursts.

On physical examination at the age of 10 years and 4 months, her weight was 35 kg (25^th^-50^th^ centile), height 138 cm (25^th^-50^th^ centile) and head circumference 60 cm (+ 5.2 SD). She had left hemihyperplasia, involving face, trunk and limbs (mainly legs) with diffusely soft and thick irregularly marbled skin and prominent capillaries and veins on the trunk, abdomen and limbs. Three small (diameter <0.5 cm) achromic spots were observed on the trunk. Her 3^rd^ right finger was significantly larger than the contralateral one and she had a bilateral proximal 4/5 cutaneous syndactyly of the 2^nd^ and 3^rd^ toes. Besides macrosomia, she had dysmorphic features including malar hypoplasia, hypertelorism (inner canthal distance >97^th^ centile, palpebral length + 1 SD), long philtrum (>97^th^ centile) and high palate, and S-shaped scoliosis.

#### Patient 2

Patient 2, the only daughter of healthy non-consanguineous parents, presented to our observation at 12 years of age with a history of macrodactyly of the right 4^th^ finger diagnosed at birth (Fig 1b). Histological examination carried out after surgical excisions performed at 3 and 5 years of age showed fibrolipomatous tissue. At 6 years of age, disease recurrence was observed, with two nodular areas (14 and 11.2 mm in diameter) revealed by ultrasound scan of the right hand soft tissues. Such areas, located adjacent to the shaft of the middle and distal phalanges, showed uneven distribution and no evidence of bone overgrowth or distortion at X-ray examination. No development of further lesions was observed at follow-up at age 18, except for a small angioma between the 4^th^ and 5^th^ right metacarpal bones, and increased volume of the already existing fibrolipomas of the right 4^th^ finger.

#### Patient 3

The proband is the first and only son of healthy, non-consanguineous parents. At birth, macrodactyly of the left 2^nd^ and 3^rd^ fingers was noted. Radiological examination showed that overgrowth involved the soft tissues and all skeletal segments (phalangeal and metacarpal bones) of the affected rays. Thus, a provisional diagnosis of apparently isolated true congenital macrodactyly was done and a periodical clinical follow-up was suggested. Over time, the two enlarged fingers showed disproportionate overgrowth. Moreover, at 6 months of age, a soft swelling appeared at the left deltoid region, which was sonographically compatible with a subcutaneous mass of adipose tissue, and, at 10 months of age, a slight overgrowth of the left arm and forearm was noted. These clinical signs suggested a diagnostic hypothesis of Proteus-like syndrome. Because of the negative functional and postural consequences of excessive volume and weight of the abnormal fingers, a surgical intervention was performed when the proband was 1 year and 3 months old to remove the 2^nd^ and 3^rd^ left fingers by amputation (Fig 1c). Skin biopsies from the affected regions were obtained during this procedure. So far, surveillance measures revealed no abnormalities. Psychomotor development seems to be normal and physical examination revealed no significant craniofacial dysmorphism.

### Molecular and biochemical analyses

Mutational analysis of *PIK3CA* exons and adjacent intronic regions was performed by Sanger sequencing methods on genomic DNA isolated from blood samples, tissue biopsies, and cultured dermal fibroblasts. No pathogenic variants were found, except for the point mutation c.241 G>A [p.E81K] in *PIK3CA* exon 1 detected in patient 1. This mutation was identified in heterozygosity in hair follicles, saliva, left leg biopsy, and left and right leg cultured dermal fibroblasts of the proband, but was undetectable in the patient's blood and in her parents (Fig 1a).

Considering the limited sensitivity of Sanger sequencing for detecting low-level mosaic mutations, we performed targeted deep sequencing of 21 selected genes involved in the PI3K/AKT/mTOR pathway both in blood and tissue/biopsy/cell culture samples of the three patients.

The mean coverage depth per sample was 1900X with a mean percentage of reads on target of 93.75%. The percentage of ROI with a minimum coverage of 200X was 97.22%, see Table C in S1 File.

This approach confirmed the presence of the c.241 G>A [p.E81K] mutation in the right and left leg biopsies of patient 1, with mutant allele frequencies of 9% and 21.5%, respectively (Fig 1d).

In both patients with a clinical diagnosis of FAO (patients 2 and 3), targeted deep sequencing analysis led to the identification of a *PIK3CA* mutation in primary fibroblasts samples only. Specifically, a c.3140 A>G [p.H1047R] mutation was identified in cultured dermal fibroblasts of patient 2 with a frequency of 9.1% (74/811 reads) (Fig 1d) and a c.3140 A>T [p.H1047L] mutation was identified in the 2^nd^ finger tissue biopsy of patient 3 with a frequency of 15.5% (116/746 reads) (Fig 1d). As shown in Fig 1b and 1c, respectively, the c.3140 A>G [p.H1047R] mutation was undetectable by Sanger sequencing, whereas the c.3140 A>T [p.H1047L] mutation was visible with this method in DNA derived from fibroblast cultures, but was absent in blood samples (data not shown).

**Fig 2:**
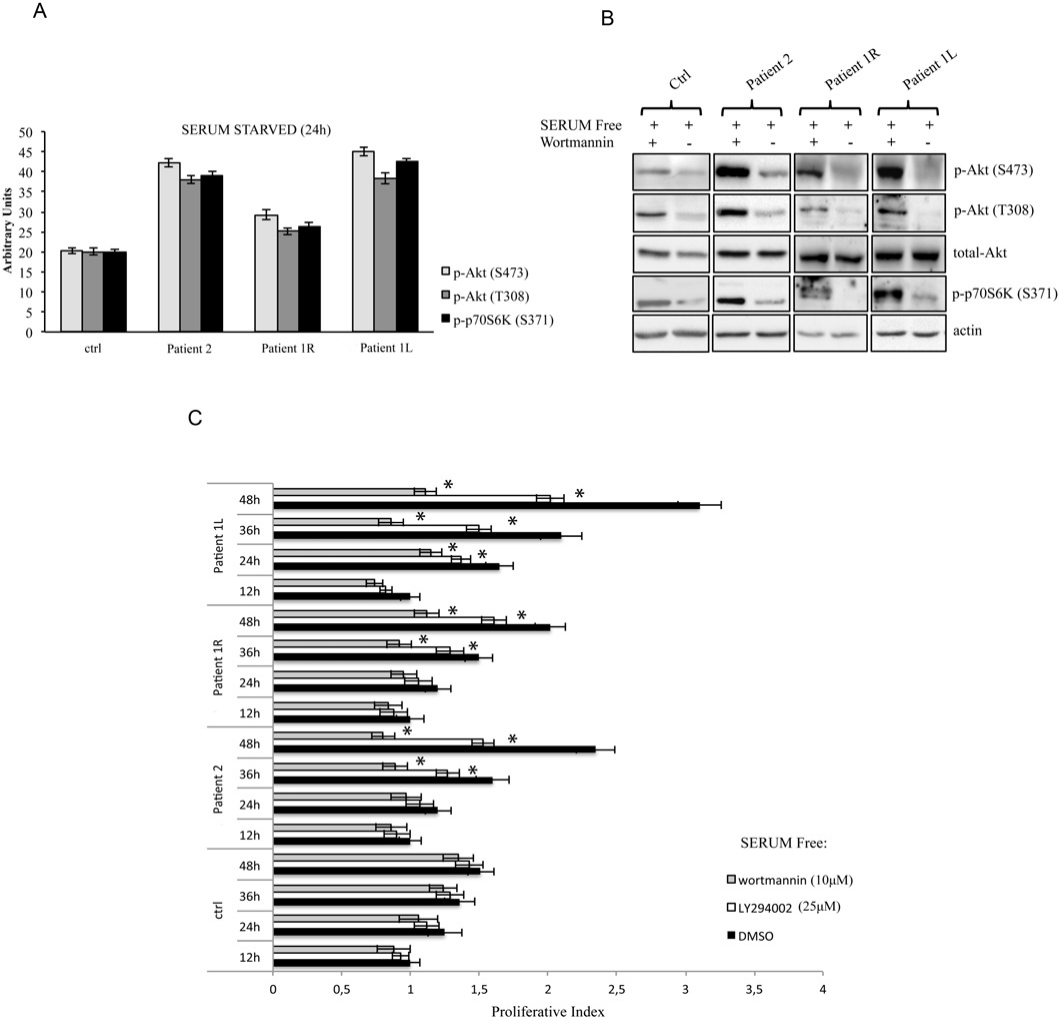
Overactivation of the PI3K/Akt pathway is abrogated by pharmacological inhibition of PI3K in all patients tested. **a** The indicated values are the result of the densitometric analysis of the phosphorylated forms of Akt and p70S6K normalized against total Akt and the loading control, respectively. The presented results are representative of at least three independent sets of experiments (bars represent standard deviation of the mean). **b** Immunoblot analysis of phospho-Akt (Ser473), phospho-Akt (Thr308), total Akt and phospho-p70S6K (Ser371) in mutant cells (fibroblasts from biopsies of FAO patient 2, and from left [L] and right [R] leg biopsies of MCAP patient 1) compared to IMR90 primary human normal fibroblasts (Ctrl). β-Actin was used as a loading control. Cells were treated with the PI3K inhibitor wortmannin (10μM) for 24 hours in the absence of growth factor stimulation. The presented results are representative of at least three independent sets of experiments. **c** Patients' affected cells are dependent on PI3K activity for proliferation. Primary fibroblasts obtained from biopsies were cultured in the presence or absence of wortmannin (10μM) and LY294002 (25μM). At the indicated time points, the proliferation index was determined using the WST-1 assay. The results were also confirmed by cell counting with trypan blue staining (data not shown). Each assay was performed in 6 replicates and the experiment was repeated six times. Statistical analysis was performed using Student’s t-tail test; *P<0.05, which was considered statistically significant (bars represent standard deviation of the mean).

*PIK3CA* mutations have been previously identified in several types of cancer, where they overactivate the PI3K/AKT/mTOR signaling in the absence of growth factors [8,9]. Thus, to functionally characterize these mutations in our patients, we cultured primary dermal fibroblasts from skin biopsies of overgrowth lesions in the absence of serum. According to our immunoblotting results (Fig 2a and 2b), mutant cells (fibroblasts from biopsy of patient 2, and from left and right leg biopsies of patient 1) showed increased levels of phosphorylated AKT at threonine 308, a residue targeted by PI3K in a direct or indirect (through PDK1) manner, when compared to IMR90 primary human normal fibroblasts. Moreover, patients’ cells showed increased phosphorylation of another AKT residue, serine 473, which is targeted by the TORC2 complex (mTOR/Rictor) in a PI3K-dependent manner [10,11]. In order to fully characterize the PI3K/AKT/mTOR cascade, we also evaluated the phosphorylation status of p70S6K, a direct substrate of the TORC1 complex (mTOR/Raptor). Our results showed that phosphorylation of serine 371, was significantly increased in overgrowing cells compared to normal healthy cells.

To complete the functional characterization of our patients’ primary fibroblasts, we treated these cells with the PI3K inhibitors wortmannin or LY294002 in the absence of growth factor stimulation. As shown in Fig 2b, overactivation of the PI3K/AKT/mTOR pathway was abrogated by pharmacological inhibition of PI3K in all patients tested as shown by the evaluation of the phosphorylation status of p-AKT and p-p70S6K. Importantly, patient-derived fibroblasts showed the ability to grow in culture even in the absence of growth factors, thus mimicking *in vivo* overgrowth features, while IMR90 primary human normal fibroblasts failed to proliferate in serum starvation conditions (Fig 2c). Moreover, growth factor-independent proliferation *in vitro* was significantly decreased by the pharmacological blockade of PI3K by both wortmannin and LY294002 in affected cells (Fig 2c), suggesting that this approach could be beneficial in overgrowth patients.

## Discussion

Our data support the hypothesis that *PIK3CA* mutations can be responsible for a wide clinical spectrum of segmental overgrowth disorders. Identical mutations have been found in phenotypically distinct disorders; however, to date any correlation with genotype is suggested but not confirmed [12]. The c.241 G>A [p.E81K] mutation identified in a patient with MCAP, patient 1, has been described previously in only one case of MCAP [2]. This mutation involves the residue 81 in the Ras-binding domain of PI3K and to our knowledge no functional analysis for this mutation has been ever performed on cells of affected patients.

According to Keppler-Nereuil [7] this patient should be categorized as PROS-A.

The *PIK3CA* c.3140 A>G [p.H1047R] mosaic mutation identified in patient 2 with overgrowth confined only to the right 4^th^ finger diagnosed at birth, has been observed in patients with KTS, CLOVES and very severe FAO, but also in cases of isolated macrodactyly [13]. On the basis of the clinical spectrum of this patient (see results), after the positive testing for PIK3CA mutation, this patient would be classified as PROS B. It is worth noting that the *PIK3CA* c.3140 A>G [p.H1047R] change is the most common cancer-associated *PIK3CA* mutation [8,9]. However, in affected tissues of overgrowth disorder patients, it is isolated and associated to a variable mutation burden, as opposed to what happens in a tumor, where it is one of several somatic mutations.

Similarly, the c.3140 A>T [p.H1047L] mutation identified in patient 3, with clinical presentation consistent with FAO, has been already described in FAO and in isolated macrodactyly type I cases, and was also reported in more than 130 human cancers in the COSMIC Database. The initial classification of this patient at referral would have been PROS B, however, after clinical follow up, he was more precisely matching the PROS A presentation.

Our functional studies on primary patient-derived fibroblasts showed overactivation of the PI3K pathway and PI3K-dependent proliferation in affected cells for all mutations analyzed. Indeed, patients’derived cells were able to grow *in vitro* in the absence of growth factors and mitogens, while displaying a significantly reduced proliferation rate upon PI3K pharmacological inhibition by both wortmannin and LY294002. Very recent reports have demonstrated that oncogenic PIK3CA mutations cause activation of PI3K pathway and proliferative advantage in cells derived from lymphatic malformations [14,15]. Our data, show that the two mutations functionally studied [H1047R, E81K] behave similarly in PROS patient-derived fibroblast.

No malignancies were identified in our patients. Although an increased cancer risk cannot be ruled out in patients with somatic *PIK3CA* mutations, data are currently very limited. This is not surprising considering that only in recent years researchers began to identify mutations associated with these disorders, which were often misdiagnosed and superficially classified as Proteus or Proteus-like syndromes [16]. Keppler-Noreuil et al. [12] described a patient diagnosed with FAO harboring a mosaic c.3140 A>T [p.H1047L] mutation that developed premalignant features of nephrogenic rests and a second patient, diagnosed with CLOVES, who had an ovarian cystadenoma; In addition, Kurek *et al*. [4] reported the case of a CLOVES patient with a mosaic c.3140 A>G [p.H1047R] mutation, who was affected by Wilms' tumor. In another report, two cases of Wilms' tumor, one case of leukemia, two cases of meningioma and one case of medulloblastoma were described, respectively, in five MCAP and one MPHH patient. However, these patients' diagnoses were merely clinical, with no molecular characterization of the underlying disorders [2].

Surgical debulking and orthopedic procedures are the only treatments currently available for patients with segmental overgrowth syndromes [16,17]. However, considering that the investigation of small molecule inhibitors of the PI3K signaling network is a promising area of oncology drug development, in the next few years these patients may well benefit from the results of completed and ongoing clinical trials on PI3K inhibitors in cancer therapy. For example, an improved wortmannin chemical analog, PX-866, displayed antitumor activity in preclinical models and is currently being tested in various clinical trials [18]. For all of these reasons, PROS patients might be regarded as very appropriate candidates for enrollment in trials based on PI3K inhibitors. In fact, they display an ideal "clean" cellular setting, as they carry only a single *PIK3CA* mutation while lacking the host of somatic mutations that usually characterize tumors and that can interfere with/modulate the complex mechanisms regulating PI3K/AKT/mTOR signaling. On the other side, they would need long-term therapies and thus should be treated with drugs with very limited and acceptable side effects.

The results presented in this study, strengthen the idea that patients with these rare and often neglected syndromes, may represent the target of future trials using clinically available inhibitors of the PI3K/AKT/MTOR pathways.

## Supporting Information

S1 File Table 1: Primers and annealing temperature for the PCR amplification of PIK3CA (NM_006218.2), AKT1 (NM_001014432.1), AKT3 (NM_005465.4), and PIK3R2 (NM_005027.2) genes. Table 2: List of PI3K/Akt/mTOR pathway genes selected for targeted deep sequencing. Table 3: Matrix Table
(DOCX)
