# Supplementary material for: Molecular and Functional Characterization of Three Different Postzygotic Mutations in PIK3CA-Related Overgrowth Spectrum (PROS) Patients: Effects on PI3K/AKT/mTOR Signaling and Sensitivity to PIK3 Inhibitors
Source: PLoS One. 2015 Apr 27;10(4):e0123092. doi: 10.1371/journal.pone.0123092 (PMC4411002; doi:10.1371/journal.pone.0123092)
Supplement: S1 File — Table 1: Primers and annealing temperature for the PCR amplification of PIK3CA (NM_006218.2), AKT1 (NM_001014432.1), AKT3 (NM_005465.4), and PIK3R2 (NM_005027.2) genes. Table 2: List of PI3K/Akt/mTOR pathway genes selected for targeted deep sequencing. Table 3: Matrix Table (DOCX) [file pone.0123092.s001.docx]

**Molecular and functional characterization of three different postzygotic mutations in *PIK3CA*-related overgrowth spectrum (PROS) patients: effects on PI3K/AKT/mTOR** **signaling and sensitivity to PIK3 inhibitors**

Daria C. Loconte ^1¶^, Valentina Grossi^1,2¶^, Cristina Bozzao^3¶^, Giovanna Forte**^4^**, Rosanna Bagnulo^1^, Alessandro Stella^1^, Patrizia Lastella^5^, Mario Cutrone^6^, Francesco Benedicenti^7^, Francesco C. Susca^1^, Margherita Patruno^1^, Dora Varvara^1^, Aldo Germani^1^, Luciana Chessa^3^, Nicola Laforgia^8^, Romano Tenconi^9^, Cristiano Simone ^1,2^ and Nicoletta Resta^1^*

*** Correspondence to:**

Prof. Nicoletta Resta, Sezione di Genetica Medica, Dipartimento di Scienze Biomediche ed Oncologia Umana, Università degli Studi di Bari ‘‘A. Moro’’,

Piazza G. Cesare 11, Bari 70121, Italy.

E-mail: [nicoletta.resta@uniba.it](mailto:nicoletta.resta@uniba.it)

Tel.:+390805593619

Fax:+390805593618

**S1 Table A. Primers and annealing temperature for the PCR amplification of *PIK3CA* (NM_006218.2), *AKT1* (NM_001014432.1), *AKT3* (NM_005465.4), and *PIK3R2* (NM_005027.2) genes.**

| **Gene** | **Exon** | **PrimerSequence** | **TA** |
| --- | --- | --- | --- |
| **PIK3CA** |  |  |  |
|  | EX 1 | F 5' gggacaaccatacatctaattcc 3' | **60** |
|  |  | R 5' ggacaacagttaagctttatgg 3' |  |
|  | EX5 | F 5' gtgtatacattagtatatacc 3' | **55** |
|  |  | R 5'gctttgttgcccaggctggtc 3' |  |
|  | EX 7 | F 5'cccattattatagagatgattg 3' | **55** |
|  |  | R 5'cctaagagatggaagaaaagc 3' |  |
|  | EX9 | F 5' catctgtgaatccagaggg 3' | **60** |
|  |  | R 5' ctccattttagcacttacctgtgac 3' |  |
|  | EX 13 | F 5' gcaaagattatttgtatactga 3' | **55** |
|  |  | R 5' ctaaacaactctgccccactg 3' |  |
|  | EX 18 | F 5' ggcctgaatcactatatttc 3' | **60** |
|  |  | R 5' gtcttgaataaattaagaacacc 3' |  |
|  | EX 20 | F 5' catcatttgctccaaactgac 3' | **55** |
|  |  | R 5' gaaagctcacctggattcc 3' |  |
| **AKT1** |  | |  |
|  | EX 2 | F 5' tagagtgtgcgtggctctca 3' | **56** |
|  |  | R 5' ctgaatcccgagaggccaa 3' |  |
| **AKT3** |  | |  |
|  | EX 8 | F 5' gcttactatttctatgtgttc 3' | **50** |
|  |  | R 5' ggatttacttcttgactc 3' |  |
|  | EX 13 | F 5' ggagctattgcctctgttcatc 3' | **55** |
|  |  | R 5' atgtgtaagagctaggac 3' |  |
| **PIK3R2** |  | |  |
|  | EX9 | F 5' cagagcagcaagactctgtctc 3' | **66** |
|  |  | R 5' gacacagggtagaggagcccg 3' |  |

**S1 Table B. List of PI3K/Akt/mTOR pathway genes selected for targeted deep sequencing.**

| **GENE** | **Location** | **Num_Amplicons** | **Overall_Coverage** | **NAME** |
| --- | --- | --- | --- | --- |
| AKT1 | chr14:105235687-105259938 | 18 | 1.0000 | Homo sapiens v-akt murine thymoma viral oncogene homolog 1 (AKT1) |
| AKT2 | chr19:40745497-40785237 | 18 | 1.0000 | Homo sapiens v-akt murine thymoma viral oncogene homolog 2 (AKT2) |
| AKT3 | chr1:243663021-244006584 | 24 | 1.0000 | Homo sapiens v-akt murine thymomaviral oncogene homolog 3 (proteinkinase B, gamma) (AKT3) |
| GAB1 | chr4:144257983-144395718 | 26 | 1.0000 | Homo sapiens GRB2-associated binding protein 1 (GAB1) |
| GAB2 | chr11:77926336-78052926 | 16 | 0.9544 | Homo sapiens GRB2-associated binding protein 2 (GAB2) |
| IRS1 | chr2:227659726-227663454 | 21 | 1.0000 | Homo sapiens insulin receptor substrate 1 (IRS1) |
| KRAS | chr12:25358180-25403854 | 10 | 0.969 | Homo sapiens v-Ki-ras2 Kirsten rat sarcoma viral oncogene homolog (KRAS) |
| MAPK8IP1 | chr11:45907047-45928016 | 17 | 0.9589 | Homo sapiens mitogen-activated protein kinase 8 interacting protein 1 (MAPK8IP1 |
| MAPKAP1 | chr9:128199673-128469513 | 18 | 0.9970 | Homo sapiens mitogen-activated protein kinase associated protein 1 (MAPKAP1) |
| MLST8 | chr16:2255178-2259418 | 10 | 0.9778 | Homo sapiens MTOR associated protein, LST8 homolog (S. cerevisiae) (MLST8) |
| MTOR | chr1:11166588-11322608 | 79 | 0.99110 | Homo sapiens mechanistic target of rapamycin (serine/threonine kinase) (MTOR) |
| PDK1 | chr2:173420779-173463862 | 20 | 0.9904 | Homo sapiens pyruvatedehydrogenasekinase, isozyme 1 (PDK1) |
| PDK2 | chr17:48172639-48188733 | 17 | 0.9888 | Homo sapiens pyruvatedehydrogenasekinase, isozyme 2 (PDK2 |
| PIK3CA | chr3:178866311-178952497 | 42 | 0.9761 | Homo sapiens phosphatidylinositol-4,5-bisphosphate 3-kinase, catalytic subunit alpha (PIK3CA) |
| PIK3R1 | chr5:67511584-67597649 | 30 | 0.9908 | Homo sapiens phosphoinositide-3-kinase, regulatory subunit 1 (alpha) (PIK3R1) |
| PIK3R2 | chr19:18263988-18281343 | 18 | 0.8770 | Homo sapiens phosphoinositide-3-kinase, regulatory subunit 2 (beta) (PIK3R2) |
| PTEN | chr10:89623195-89728532 | 16 | 0.9964 | Homo sapiens phosphatase and tensin homolog (PTEN) |
| PTPN11 | chr12:112856536-112947717 | 22 | 0.9690 | Homo sapiens protein tyrosine phosphatase, non-receptor type 11 (PTPN11) |
| RAPTOR | chr17:78518625-78940173 | 47 | 0.9943 | Homo sapiens regulatory associated protein of MTOR, complex 1 (RPTOR) |
| RICTOR | chr5:38938022-39074501 | 77 | 0.9210 | Homo sapiens RPTOR independent companion of MTOR, complex 2 (RICTOR) |
| THEM4 | chr1:151843343-151882361 | 8 | 0.9252 | Homo sapiens thioesterase superfamily member 4 (THEM4) |

**S1 Table C. Matrix Table**

|  | **SM 122/08**  **blood** | **SM 122/08**  **cell culture** | **EW 1162/13 blood** | **EW 1162/13 biopsy** | **EB 1167/13**  **Right cell culture** | **EB 1167/13**  **Left cell culture** | **LG 274/14 blood** | **LG 274/14**  **cell culture** |
| --- | --- | --- | --- | --- | --- | --- | --- | --- |
| **Total Reads** | 1380556 | 1666507 | 728641 | 722644 | 863458 | 846565 | 1434976 | 1305023 |
| **% Reads On Target** | 96,35 | 96,54 | 91,46 | 92,03 | 96,59 | 96,62 | 96,47 | 95,85 |
| **% Uniformity** | 94,92 | 94,83 | 95,98 | 95,47 | 94,44 | 94,29 | 95,26 | 95,31 |
| **Mean Read Lengthbp** | 149 | 150 | 142 | 143 | 154 | 155 | 153 | 151 |
| **MeanROI Coverage** | 2401,06 | 2904,19 | 1202,89 | 1200,39 | 1505,51 | 1476,44 | 2498,75 | 2257,76 |
| **%ROI Covered>200X** | 97,65 | 97,83 | 96,57 | 96,57 | 95,31 | 95,31 | 98,19 | 98,01 |
| **N Variants** | 61 | 62 | 53 | 54 | 56 | 56 | 57 | 57 |
